# Angelman syndrome-derived neurons display late onset of paternal *UBE3A* silencing

**DOI:** 10.1038/srep30792

**Published:** 2016-08-03

**Authors:** Jana Stanurova, Anika Neureiter, Michaela Hiber, Hannah de Oliveira Kessler, Kristin Stolp, Roman Goetzke, Diana Klein, Agnes Bankfalvi, Hannes Klump, Laura Steenpass

**Affiliations:** 1 Institute of Human Genetics, University Hospital Essen, University of Duisburg-Essen, Hufelandstr. 55, 45147 Essen, Germany; 2 Institute for Transfusion Medicine, University Hospital Essen, University of Duisburg-Essen, Hufelandstr. 55, 45147 Essen, Germany; 3 Helmholtz Institute for Biomedical Engineering, Stem Cell Biology and Cellular Engineering, RWTH Aachen University, Pauwelsstr. 20, 52074 Aachen, Germany; 4 Institute for Cell Biology, University Hospital Essen, University of Duisburg-Essen, Hufelandstr. 55, 45147 Essen, Germany; 5 Institute of Pathology, University Hospital Essen, University of Duisburg-Essen, Hufelandstr. 55, 45147 Essen, Germany

## Abstract

Genomic imprinting is an epigenetic phenomenon resulting in parent-of-origin-specific gene expression that is regulated by a differentially methylated region. Gene mutations or failures in the imprinting process lead to the development of imprinting disorders, such as Angelman syndrome. The symptoms of Angelman syndrome are caused by the absence of functional UBE3A protein in neurons of the brain. To create a human neuronal model for Angelman syndrome, we reprogrammed dermal fibroblasts of a patient carrying a defined three-base pair deletion in *UBE3A* into induced pluripotent stem cells (iPSCs). In these iPSCs, both parental alleles are present, distinguishable by the mutation, and express *UBE3A*. Detailed characterization of these iPSCs demonstrated their pluripotency and exceptional stability of the differentially methylated region regulating imprinted *UBE3A* expression. We observed strong induction of *SNHG14* and silencing of paternal *UBE3A* expression only late during neuronal differentiation, *in vitro*. This new Angelman syndrome iPSC line allows to study imprinted gene regulation on both parental alleles and to dissect molecular pathways affected by the absence of UBE3A protein.

The epigenetic process of genomic imprinting is controlled by differentially methylated regions (DMRs) which results in parent-of-origin-dependent gene expression. Imprinted germ line DMRs are exceptional in three ways: First, they can be properly and entirely established only during female or male gametogenesis. Second, they are protected from demethylation during the global wave of methylation erasure during early embryonic development. Third, methylation of the DMR is invariably preserved during cell division, present in every cell type and independent of gene expression activity^1^,^2^. Deletion of a DMR or disturbances in its methylation leads to imprinting disorders, such as Angelman syndrome (AS), exhibiting typical symptoms like absence of speech, movement disorders, happy demeanor and developmental delay^3^. The cause for these symptoms is the absence of a functional UBE3A protein in the brain^4^,^5^. The *UBE3A* gene is part of the imprinted Prader-Willi/Angelman syndrome locus (PWS/AS locus) on chromosome 15q11q13. At this locus, only the maternal allele is methylated at a CpG island, which encompasses the promoter and exon 1 of the *SNRPN* gene and represents the DMR of the locus. This DMR is termed PWS-SRO, which stands for Prader-Willi syndrome shortest region of deletion overlap^6^. DNA methylation silences the protein-coding gene *SNURF*/*SNRPN* and several non-coding RNA genes on the maternal allele^6^ (Fig. 1A). *SNURF*/*SNRPN*, the non-coding *SNORD* gene clusters and the long non-coding RNA, *SNHG14* (alternatively named *UBE3A-ATS*) are expressed from the non-methylated paternal allele. In the brain, *SNHG14* overlaps the entire *UBE3A* gene and promoter in antisense direction, thereby silencing *UBE3A* expression^7^. As a consequence, this results in brain-specific monoallelic *UBE3A* expression from the maternal allele. Hence, *UBE3A* is vulnerable to mutations occurring on the maternal chromosome 15. So far, large deletions up to several megabases, imprinting defects, paternal uniparental disomy or mutations in the *UBE3A* gene itself have been described as molecular cause for AS^3^. The different types of mutations correlate with gradual differences in the severity of the disorder. Large deletions result in loss of several other genes in the same region, and these patients typically present with a more severe phenotype than patients carrying point mutations affecting the *UBE3A* gene alone^8^.

As AS is caused by a lack of UBE3A activity in the brain, access to neurons is needed to study its function at the molecular level. Therefore, the generation of induced pluripotent stem cells (iPSCs) from patient cells and their subsequent directed differentiation into neurons provide a valuable tool for AS research^9^. The generation of iPSCs from patients with Angelman syndrome has been described by Chamberlain *et al*.^10^. These AS iPSC lines carry large deletions on their maternal chromosome, resulting in loss of about 28 genes from *NIPA1* to *OCA2*. This makes it difficult to specifically address the role of *UBE3A* in neuronal function and disease development.

Here we report the establishment and thorough characterization of a new iPSC line (AS_∆3) of a previously described patient with AS, harboring a defined three-base pair deletion within the maternally inherited *UBE3A* allele^11^. The encoded protein is predicted to lack amino acid G538 (based on NM_130838). Using computer modeling of the mutant protein based on the X-ray structure of the wild-type enzyme, a local destabilization around the catalytic cleft of UBE3A was proposed, likely impairing the binding of substrates^11^. The new iPSC line complements the existing AS iPSCs carrying large chromosomal deletions. It will facilitate the specific attribution of effects observed during neuronal differentiation to the defective *UBE3A* gene. This will contribute to a deeper understanding of imprinting mechanisms and AS itself.

## Results

We reprogrammed primary dermal fibroblasts isolated from a female patient with AS harboring a three-base pair deletion in exon 4 of the *UBE3A* gene (accession NM_130838)^11^, and from a normal healthy control person. The reprogramming efficiencies (i.e. the number of isolated colonies per transduced cell number) were similar for patient and control person-derived fibroblasts, ranging from 0.005 to 0.05 percent (Supplementary Table S1). For quality and potency characterization, eight AS_∆3 and seven control iPSC clones were expanded and established as independent lines. As determined by Southern blot analysis, the number of integration sites ranged from one to five in independent clones (Supplementary Fig. S1). For further analysis, only clones containing single vector integrations were chosen: patient-derived AS_Δ3 iPSC clones #B1, #D, #H and #P, and healthy control-derived iPSC clones #42 and #645. The identity of parental fibroblast cells and derived iPSCs was confirmed by high resolution HLA typing (Supplementary Table S2) and the presence of the three-base pair deletion in exon 4 of the *UBE3A* gene in AS_∆3 iPSCs was confirmed by sequencing (Supplementary Fig. S1). Karyotype analysis revealed a normal female karyotype for three of the four patient lines and both control lines (Supplementary Fig. S1). Patient #H carries an additional marker chromosome present in all metaphases analyzed (Supplementary Fig. S1). This marker chromosome was identified as an isochromosome 12p. Gain of chromosome 12 or i12p has been reported as frequent chromosomal abnormality in hESCs and iPSCs, being probably associated with a proliferation advantage of cells^12^.

**Figure 1:**
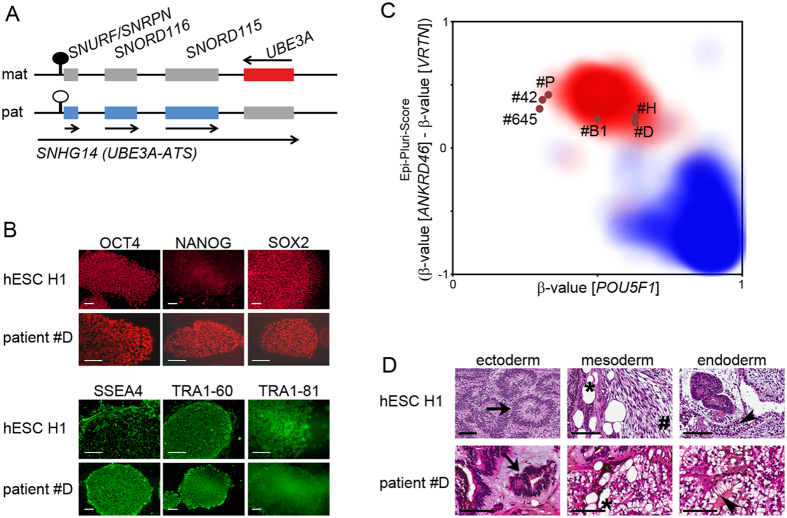
Generation of AS_∆3 iPSCs. (**A)** Schematic and simplified representation of the Prader-Willi/Angelman syndrome locus and its expression status in the brain. Red: maternally expressed genes, blue: paternally expressed genes, grey: non-expressed genes. Lollipop indicates the DMR (black: methylated, white: not methylated). Arrows indicate direction of expression. Scheme is not drawn to scale. (**B)** Immunofluorescence for nuclear (red) and surface (green) antigens showing expression of pluripotency-associated proteins in patient #D and hESC H1 as a reference. Scale bars indicate 100 μm. (**C)** Epi-Pluri-Score analysis of generated iPSCs. Clouds represent areas of reference pluripotent (red) or somatic (blue) cells. The six iPSC clones analyzed are shown as dots localizing to the pluripotent area. (**D)** H&E stained histology of immature teratomas formed by hESC H1 and patient #D. Derivatives of all three germ layers can be identified: neuroectodermal rosettes (arrow); mesodermal loose immature mesenchyme (hash) and fat cells (asterisk), and endodermal glandular tissue with clear and goblet cells (arrowhead). Scale bars indicate 100 μm.

For potency testing, expression of pluripotency markers was determined by different methods. For all assays, human embryonic stem cells (hESCs) H1 were used as a reference for pluripotency. Staining for alkaline phosphatase activity showed expression of the enzyme in all six iPSC clones (Supplementary Fig. S2). Expression of the nuclear proteins OCT4, NANOG, SOX2 and the cell surface antigens SSEA4, TRA1-60 and TRA1-81 was demonstrated by immunofluorescence and, for the surface antigens, by flow cytometry (Fig. 1B, Supplementary Figs S2 and S3). Expression of genes indicative of pluripotency, like endogenous *POU5F1* (encoding OCT4), *KLF4*, *SOX2, MYC, NANOG*, *DNMT3B*, *LIN28* and *REX1* was evaluated by real-time qPCR (Supplementary Fig. S4). All iPSCs expressed these genes at levels similar to hESC H1. In contrast, their expression was not detected in the parental fibroblasts, except for *KLF4* and *MYC*. In addition, we used TaqMan human stem cell pluripotency arrays to assess expression of 96 genes indicative of pluripotency, stemness and differentiated cell lineages. Applying cluster analysis, hESC H1, AS_∆3 and control iPSCs clustered separately from the two parental fibroblast lines, indicating successful reprogramming (Supplementary Fig. S4, Supplementary Table S3). As molecular test for pluripotency, we employed Epi-Pluri-Score analysis, using the DNA methylation levels of three single CpG sites indicative of differentiated and pluripotent cells: pluripotent cells exhibit high levels of DNA methylation at *ANKRD46* and low levels at *VRTN* and *POU5F1*^13^. All six iPSCs generated in this study, four AS_∆3 and two control clones, mapped to the upper middle area of the plot representative of reference pluripotent cells (Fig. 1C). As the most stringent test for pluripotency of human iPSCs, teratoma assays were performed. Mice were injected with reprogrammed cells of patient #B1, #D and #P, control #645 and also with hESC H1 as a positive control. Tumors formed within three to 10 weeks with 100 percent efficiency (Supplementary Table S4). Histomorphologic analysis revealed that all tumors were immature teratomas containing derivatives of all three germ layers (Fig. 1D, Supplementary Fig. S5). In summary, all tests applied indicated that the newly generated patient and control iPSCs are pluripotent and fully reprogrammed.

**Figure 2:**
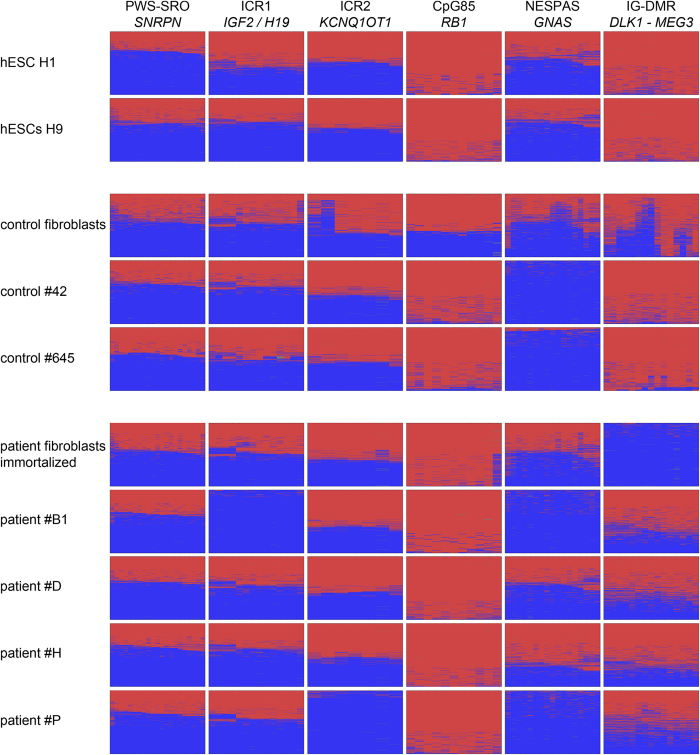
Epigenetic stability of six imprinted DMRs. DNA methylation was analyzed by deep bisulfite amplicon sequencing. The heatmaps show each CpG site in columns and individual sequenced reads in rows. Red: methylated, blue: unmethylated. Most heatmaps display a 50% methylation level, which is expected for imprinted DMRs in diploid cells. Exceptions are a loss of methylation at ICR1 (#B1), ICR2 (#P) and NESPAS (#42, #645, #B1, #P). CpG85 shows consistent gain of methylation, except for the control fibroblast sample. IG-DMR exhibits hypermethylation in both hESC lines, #42 and #645 and hypomethylation in the immortalized patient-derived fibroblast sample.

AS_∆3 iPSCs were generated as a tool for modeling the imprinting disorder AS. For proper imprinted gene expression of the AS gene *UBE3A*, epigenetic stability of the PWS-SRO is mandatory. Using deep bisulfite amplicon sequencing, analysis of DNA methylation was performed in parental fibroblasts, hESCs H1 and H9 and the iPSCs generated in this study at the PWS-SRO and five additional germ line DMRs of imprinted gene clusters or genes: the IG-DMR at the *DLK1 - MEG3* locus, the CpG85 DMR at the *RB1* gene, the NESPAS DMR at the *GNAS* locus, and the ICR2 and ICR1 DMRs (Fig. 2, Supplementary Table S5). DNA methylation of the latter two regulates expression at the Beckwith-Wiedemann syndrome locus and the *IGF2*/*H19* locus, respectively. Imprinted DMRs are expected to show a level of about 50% DNA methylation in all types of diploid human cells. This level was observed consistently only for the PWS-SRO in all samples tested. The CpG85 at the *RB1* gene showed consistent hypermethylation to a level of almost 100%, except for the fibroblasts derived from the control person. The PWS-SRO and the CpG85 represent the extremes of our analysis, being the most stable and unstable DMRs, respectively. The DMRs ICR1, ICR2 and NESPAS mainly exhibited the expected level of 50% methylation, but showed hypomethylation in one (ICR1, ICR2) and four (NESPAS) clones. The IG-DMR was reported to be prone to a gain of methylation in human pluripotent cells^14^,^15^, which we observed in the two hESC lines and both control iPSC clones, but not in the patient-derived clones. In contrast, immortalized fibroblast cells of the patient showed a loss of methylation at the IG-DMR, which was not confirmed in fibroblasts of healthy control persons and blood lymphocytes of the patient (Supplementary Fig. S6). For comparison, we analyzed the methylation status in the previously published AS iPSCs carrying a large type I deletion of the whole PWS/AS locus on the maternal allele (line AGI-0) and control cells (line MCH2-10). As expected, in AGI-0 the PWS-SRO was completely unmethylated as only the paternal allele is present (Supplementary Fig. S6). Methylation levels at the ICR1, ICR2 and NESPAS were normal, whereas the CpG85 and the IG-DMR showed a gain of methylation. Results for MCH2-10 iPSCs were comparable, except for normal 50% methylation at the PWS-SRO and a loss of methylation at the NESPAS (Supplementary Fig. S6).

**Figure 3:**
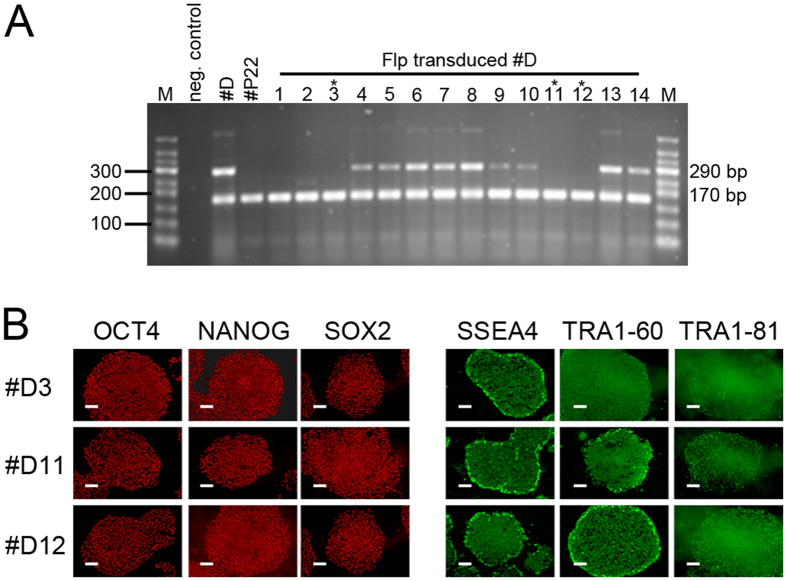
Virus excision from patient #D iPSCs. (**A)** PCR screening for successful virus excision indicated by absence of the 290 bp product, the 170 bp product serves as internal control. Clones marked by an asterisk (#D3, #D11 and #D12) were analyzed further. (**B)** Positive immunofluorescence of three excised clones of parental patient #D for pluripotency-associated nuclear (OCT4, NANOG, SOX2) and surface (SSEA4, TRA1-60, TRA1-81) antigens. Scale bars indicate 100 μm.

For the generation of iPSCs from dermal fibroblasts, we used a self-inactivating lentiviral reprogramming vector, which contains two FRT sites in the ∆U3 regions within the LTRs that allow for excision of the viral vector by Flp recombinase after successful reprogramming^16^,^17^. To eliminate the risk of reactivation of the integrated vector, which could lead to inhibition of differentiation^18^, we delivered Flp recombinase into patient #D and #P and control #42 and #645 by lentiviral-mediated protein transfer to excise the reprogramming vector. Successful excision was screened for by PCR (Fig. 3A, Supplementary Fig. S7). Two to three subclones were analyzed of each parental iPSC clone and all maintained their pluripotency characteristics, as determined by expression analysis of alkaline phosphatase and selected pluripotency-associated genes (Fig. 3B, Supplementary Fig. S7). Pluripotency of the excised subclone #P22 was also functionally proven by successful teratoma formation in mice (Supplementary Fig. S5).

Imprinted expression of *UBE3A* is observed only in the brain. To study its function and regulation, neurons are needed. Only AS_Δ3 #D iPSCs passed all quality and pluripotency assessments and were chosen for *in vitro* differentiation into mature neurons. The generated AS_Δ3 iPSCs contain both parental *UBE3A* alleles, whose expression can be distinguished by single-nucleotide primer extension analysis using the three-base pair deletion in exon 4 (accession NM_130838) as a SNP. This offers the unique possibility to observe silencing of the paternal allele and persistent maternal *UBE3A* expression in the same cell.

**Figure 4:**
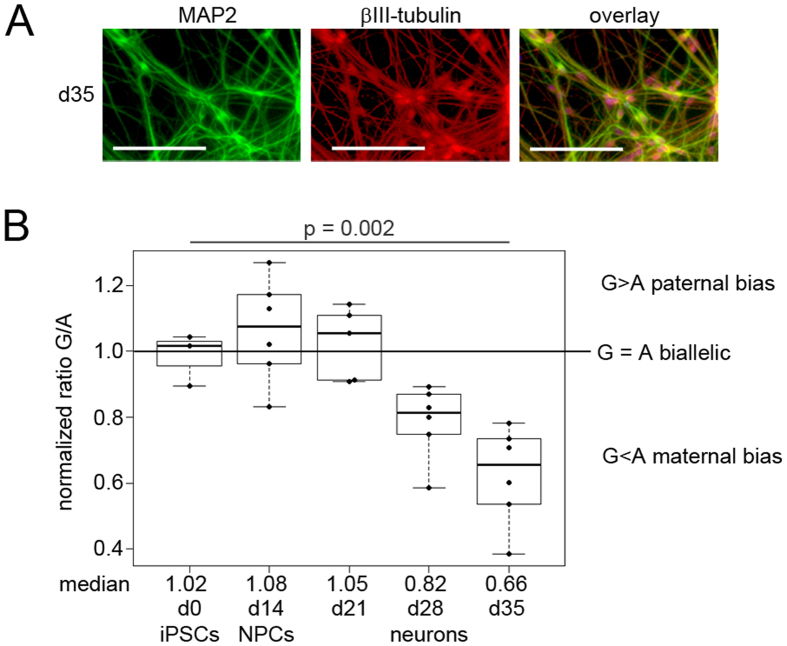
Paternal *UBE3A* expression is silenced late in neuronal differentiation. (**A)** Neurons at d35 of differentiation stained positive for MAP2 and βIII-tubulin. Scale bars indicate 100 μm. (**B)** SNaPshot analysis of the allelic expression ratio of the paternal (G) and the maternal (A) allele of *UBE3A*. The G/A ratio decreased at day 28 and day 35 in the time course of neuronal differentiation, indicating that silencing of the paternal allele is a late event.

Successful differentiation into neurons within 35 days was confirmed by staining for MAP2 and βIII-TUBULIN, which are both markers of terminally differentiated, postmitotic neurons (Fig. 4A, Supplementary Fig. S8). Using qPCR, we observed a consistent upregulation of the early neural marker *NESTIN* and the neural progenitor marker *PAX6* from day 14 on (Supplementary Fig. S8). Expression of βIII-TUBULIN was detectable at day 14 and increased up to day 35. Total expression of *UBE3A* increased during differentiation and a slight induction of the brain-specific non-coding RNA *SNHG14* was observed at day 14 which subsequently strongly increased during terminal differentiation (Supplementary Fig. S8). To determine at which time point the expression of *SNHG14* induces imprinted expression of *UBE3A*, single-nucleotide primer extension analysis in differentiated AS_∆3 iPSCs was employed. In the analysis, the paternal wildtype allele is represented by a G. The maternal allele carrying the mutation is represented by an A. As expected, undifferentiated iPSCs showed biallelic expression of *UBE3A*, indicated by a G/A ratio of about 1. Expression of *UBE3A* remained biallelic up to day 21 of neuronal differentiation. Starting with day 28, a reduction in the G/A ratio was observed, indicating higher expression from the maternal (A) than from the paternal (G) allele. At day 35 of differentiation, the mean G/A ratio, was about 0.6. This can be attributed to the onset of *UBE3A* silencing by the expression of *SNHG14* from the paternal allele. Despite the onset of paternal *UBE3A* silencing in neurons, the total level of UBE3A protein was comparable between undifferentiated iPSCs and neurons at d35 (Supplementary Fig. S9).

## Discussion

We present here the generation and detailed characterization of iPSCs from a patient with AS carrying a mutation in the *UBE3A* gene. With these cells it is possible to follow the onset of imprinted *UBE3A* expression by analyzing expression of both parental alleles in one cell. Deep bisulfite amplicon sequencing was used to determine the epigenetic stability of DMRs at six imprinted gene loci, showing that only the PWS-SRO, the DMR of the PWS/AS locus, was stable in all clones analyzed. Excision of the reprogramming vector did not compromise pluripotency, resulting in the derivation of AS_∆3 iPSCs with only a minimum of ectopic DNA. During differentiation of iPSCs into neurons, we observed strong induction of *SNHG14* expression, which accompanied silencing of paternal *UBE3A* expression. Our results indicate that silencing of paternal *UBE3A* expression by *SNHG14* is a late event during neuronal differentiation.

DMRs are the regulators of imprinted gene expression and in general their epigenetic status is stable in established hESC lines^19^. However, failures in imprint maintenance during reprogramming to iPSCs or due to prolonged time in culture have been widely discussed and described^14^,^15^. It is therefore mandatory for iPSCs to be used as models for imprinting disorders to analyze the status of DNA methylation of at least the DMR addressed in the study. Using deep bisulfite amplicon sequencing, we demonstrated that the PWS-SRO was the only DMR showing stable differential DNA methylation in all pluripotent cells and fibroblasts analyzed (Fig. 2, Supplementary Fig. S6). As expected, the AS iPSC line AGI-0, carrying a large type I deletion on the maternal chromosome 15, showed only unmethylated reads resulting from the retained paternal allele. This exceptional stability of differential DNA methylation at the PWS-SRO during reprogramming is in agreement with earlier reports^10^,^15^. In contrast, we observed a loss of differential DNA methylation at the germ line DMRs ICR1, ICR2 and NESPAS in at least one sample (Fig. 2, Supplementary Fig. S6). The IG-DMR has been described to be susceptible to hypermethylation in human pluripotent cells and in agreement with that we report a gain of DNA methylation in 7 of 12 pluripotent samples^14^,^15^. However, we observed a loss of DNA methylation at the IG-DMR in the immortalized fibroblasts of the patient, which was not seen in another three fibroblast samples analyzed (Fig. 2, Supplementary Fig. S6). We therefore assume that this loss of methylation is the result of immortalization, as changes in imprinted gene expression and imprint methylation have been observed in immortalized lymphoblastoid cell lines and fibroblasts^20^,^21^. The CpG85 was the least stable DMR, showing maintenance of differential methylation only in two fibroblast samples from independent healthy control subjects and a blood sample from the patient (Supplementary Fig. S6).

Overall, our observations indicate a non-random pattern of deviating methylation at most ICRs: the CpG85 and the IG-DMR exhibited only a gain of methylation, whereas the NESPAS and the ICR2 showed only a loss of methylation. The exception was the ICR1, where we observed one loss and one gain of methylation. We did not observe preferred combinations of DMRs in clones having more than one aberrantly methylated DMR. The molecular mechanisms predisposing DMRs to a gain or a loss of methylation in hESCs or iPSCs are not clear yet. Both types of cells are derived from somatic cells, which are expected to carry stable methylation imprints^1^,^2^, like we observed for the fibroblast sample of the control person: except for the CpG85 at *RB1*, all imprinted DMRs displayed the expected pattern of 50% DNA methylation (Fig. 2). This leads to the assumption that aberrant imprint methylation in hESCs and iPSCs is due to a failure of imprint maintenance either during reprogramming or during cultivation of fibroblasts and the derived iPSCs or hESCs^14^,^15^,^22^. Indeed, we observed a gain of methylation at ICR1 in a sample of hESCs H9 at a later passage (Fig. 2, Supplementary Fig. S6). Differences in stability of paternal and maternal imprints have been discussed^22^ but our observation of consistent gain of methylation at both maternal and paternal DMRs, the CpG85 and the IG-DMR, respectively, is not in line with this hypothesis.

Angelman syndrome is caused by the absence of functional UBE3A protein in the brain. Although microcephaly is a frequent finding in patients with AS and delayed myelinization was repeatedly observed in infant patients, gross structural abnormalities of their brains have not been reported^3^,^23^. The normal brain structure of patients with AS suggests that the defect in UBE3A protein expression or function does not restrict neuronal development and differentiation, but might interfere with neuron function. Since no patient-derived consecutive brain samples can be obtained at different stages of development, iPSCs and their subsequent differentiation into neurons are a valuable tool for the understanding of imprinting regulation at the PWS/AS locus during neuronal development. We differentiated AS_Δ3 iPSCs into mature postmitotic neurons and monitored allelic expression of *UBE3A* using the three-base pair deletion as a SNP (Fig. 4, Supplementary Fig. S8). We observed strong upregulation of *SNHG14* and a decrease in the ratio of paternal to maternal *UBE3A* expression only late during neuronal differentiation. This indicates that *UBE3A* imprinted expression possibly occurs only in postmitotic neurons, as it was described in mice^24^,^25^. It is noteworthy that *UBE3A* expression per se shows an increase in neurons, despite the silencing of the paternal allele, indicating enhanced expression from the maternal allele in neurons. This is in line with our observation of stable UBE3A protein levels in neurons, which has also been described by Chamberlain *et al*.^10^.

The AS_Δ3 iPSCs generated in this study complement the AS iPSCs carrying large deletions on the maternal chromosome that were generated by Chamberlain *et al*.^10^. This new iPSC line has the advantage that it does not carry large genomic deletions and will express a UBE3A protein that differs by only one missing amino acid from its normal counterpart. This opens the possibility to assign aberrant protein-protein interactions and functional processes specifically to the defective UBE3A protein. In addition, the mechanisms leading to *UBE3A* imprinted expression can be studied on both parental alleles in an almost normal cellular environment. Further characterization of neuronal differentiation using the two available AS iPSC lines will help to define the time and the cell type at and in which silencing of paternal *UBE3A* expression occurs. Such analyses would greatly benefit from their verification in iPSC lines generated from additional patients with AS. The knowledge gained by neuronal differentiation of AS iPSCs is urgently needed to develop efficient potential therapeutic approaches for AS.

## Methods

An additional and extended Materials and Methods section is included in the supplement.

### Statements

The use of hESCs H1 and H9 is in compliance with the German Stem Cell Law and covered by permission AZ:3.04.02/0099.

This study on reprogramming human dermal fibroblasts into induced pluripotent stem cells and all applied experimental protocols were approved by the local ethics committee of the University Hospital Essen. All applied methods were performed in accordance with the approved guidelines. We confirm that informed consent for skin biopsies were obtained from all subjects, that is the healthy control persons and the legal representative of the patient with Angelman syndrome.

The teratoma formation assays performed in this study were carried out in strict accordance with the recommendations of the Guide for the Care and Use of Laboratory Animals of the German Government and it was approved by the Committee on the Ethics of Animal Experiments of the responsible authorities (*Landesamt für Natur, Umwelt und Verbraucherschutz*, LANUV AZ 8.87-50.10.37.09.187 and AZ 84-04.04.2013.A350).

### Cell culture, reprogramming and virus excision

hESC and iPSC lines were kept on proliferation-arrested, CF1 feeder cells in KSR medium (DMEM/F12 Glutamax, 20% knockout serum replacement, 4 ng/μl bFGF, 50 U/ml Penicillin/Streptomycin, non-essential amino acids, 40 ng/ml heparin), or feeder-independent in mTeSR1 on Vitronectin-coated tissue-culture dishes. Cells were kept at 37 °C and 5% CO_2_ in a humidified incubator. Skin biopsies were cultivated *in vitro* and obtained fibroblasts were reprogrammed using a lentiviral vector co-expressing the coding sequences of *POU5F1*, *KLF4*, *MYC* and *SOX2* together with the gene encoding tomato fluorescent protein^17^. Seven days post transduction, cells expressing the tomato fluorescent protein were isolated by flow cytometric cell sorting. Colonies were picked approximately four weeks post transduction and expanded on irradiated feeder cells in KSR medium. Virus excision was mediated by direct protein transduction of Flp recombinase into iPSCs^16^. Isolated single clones were screened by PCR for absence of integrated virus genome.

### Immunofluorescence

Immunofluorescent antibody staining of adherent iPSC colonies was conducted using the StemLite Pluripotency kit from Cell Signaling Technology, providing antibodies for the nuclear antigens OCT4, NANOG, SOX2 and surface antigens TRA1-60, TRA1-81 and SSEA4. Stainings were done according to manufacturer’s instructions. Antibodies and dilutions are listed in Supplementary Table S7.

### Teratoma formation assay

Mice were kept under standard conditions (12 hours light and dark cycle, food and water ad libitum) in the Central Animal Facility of the University Hospital Essen. Cells were resuspended in DMEM/F12 supplemented with 50% growth-factor reduced Matrigel and 1 × 10^6^ cells/ml were injected subcutaneously into both hind limbs of immunodeficient *NMRI nu*/*nu* mice (*Harlan Laboratories)*. Mice were monitored daily for teratoma growth. Tumors were explanted when they reached a critical size, when skin lesions appeared, or latest 67 days after injection. For teratoma histopathology, 5 μm paraffin cross-sections were stained with hematoxylin and eosin.

### Deep bisulfite sequencing and Epi-Pluri-Score analysis

Deep bisulfite sequencing was performed on the 454 GS junior platform (Roche) as described previously^26^. Data analysis was done using the Amplikyzer software^27^ and results are depicted as methylation heatmaps showing single CpG sites in columns and single reads in rows. The percentage of overall methylation is given in Supplementary Table S6. Amplicon-specific tagged primer sequences are given in Supplementary Table S8, as well as an example for a MID-primer pair. For the Epi-Pluri-Score analysis, pyrosequencing of three CpGs of interest (cg 23737055 in *ANKRD46*, cg22247240 in *VRTN*, cg13083810 in *POU5F1*) was performed and analyzed as described by Lenz *et al*.^13^. The Epi-Pluri-Score is calculated as the difference of β-value(*ANKRD46*) minus β-value(*VRTN*). A positive value is indicative of pluripotent cells, a negative value of differentiated cells. The Epi-Pluri-Score is plotted against the β-value measured for *POU5F1*. In the plot, reference sets of 265 pluripotent and 1,951 somatic cells are indicated as clouds. Primer sequences are listed in Supplementary Table S8.

### Neuronal differentiation

Neuronal differentiation was started from a monolayer culture of iPSCs at 50–60% confluency by changing to induction medium (50% DMEM/F12/Glutamax, 50% Neurobasal, 0.5% N2, 1% B27 without retinoic acid, 20 μM SB431543, 100 nM LDN193189). Induction medium was changed every other day. At day 10, medium was changed to neural expansion medium (DMEM/F12/Glutamax, 1% N2, 2% B27 without retinoic acid, 20 ng/ml FGF2, 20 ng/ml EGF, 200 μM ascorbic acid). At day 13, neural progenitor cells were enriched by positive selection using magnetic anti-PSA-NCAM MicroBeads (Miltenyi Biotec). Cells were passaged every three to four days as single-cell suspension. Terminal differentiation was induced by plating a single cell suspension onto culture vessels coated with poly-D/L-ornithine and laminin at a density of 3 × 10^4^/cm^2^ in terminal differentiation medium (Neurobasal, 2% B27, 2 mM Glutamax, 1% non-essential amino acids, 200 μM ascorbic acid, 20 ng/ml BDNF, 20 ng/ml GDNF). 50% of medium was changed every other day up to the final time point of differentiation. Cells were harvested at the day of seeding (undifferentiated iPSCs), at day 14 (neural progenitor cells) and days 21, 28 and 35 during the terminal differentiation phase for RNA extraction and immunofluorescence.

### SNaPshot analysis

The ABI Prism SNaPshot ddNTP Primer Extension Kit (Life Technologies) was used to determine allelic ratios of mRNA transcripts (after reverse transcription into cDNA) following the manufacturer’s instructions. Genomic DNA of the respective iPSC clones was used as a reference. The reaction products were analyzed on an ABI 3130XL sequencer and electropherograms were analyzed using Gene Mapper 4.0 software (Applied Biosystems, Life Technologies). The “area” value in the Gene Mapper graphical output was used as indicator for the amount of PCR products, amplified from cDNA, extended by a G (product of the paternal allele) or an A (product of the maternal allele). Next, the G/A ratio was calculated and normalized to the G/A ratio of genomic DNA. Analysis was done in three biological and two technical replicates. All data points are included in the bee swarm boxplots, which were calculated using the respective packages provided by The R Project for Statistical Computing. In the boxplots, the median and quartiles are indicated. The p-values were calculated in R using Welch’s unequal variances t-test. Primer sequences are listed in Supplementary Table S8.

## Additional Information

**How to cite this article**: Stanurova, J. *et al*. Angelman syndrome-derived neurons display late onset of paternal *UBE3A* silencing. *Sci. Rep*. **6**, 30792; doi: 10.1038/srep30792 (2016).

## Supplementary Material

Supplementary Information
