# Corrigendum: Angelman syndrome-derived neurons display late onset of paternal *UBE3A* silencing

**DOI:** 10.1038/srep46952

**Published:** 2018-03-08

**Authors:** Jana Stanurova, Anika Neureiter, Michaela Hiber, Hannah de Oliveira Kessler, Kristin Stolp, Roman Goetzke, Diana Klein, Agnes Bankfalvi, Hannes Klump, Laura Steenpass

Scientific Reports 6: Article number: 3079210.1038/srep30792; published online: 08 03 2016; updated: 03 08 2018

This Article contains typographical errors. In the Results section,

“We reprogrammed primary dermal fibroblasts isolated from a female patient with AS harboring a three-base pair deletion in exon 4 of the *UBE3A* gene (accession NM_130838)^11^, and from a normal healthy control person.”

should read:

“We reprogrammed primary dermal fibroblasts isolated from a female patient with AS harboring a three-base pair deletion in exon 5 of the *UBE3A* gene (accession NM_130838)^11^, and from a normal healthy control person.”

In Table S8 of the Supplementary Information file, the sequence of the forward primer ‘SNHG14_RT17_F’ for ‘qRT-PCR neuronal differentiation’,

“cttgagtattccggaagtaaaagc”

should read:

“ctcttcttgcagtttacaacgg”

