# Supplementary material for: Angelman syndrome-derived neurons display late onset of paternal UBE3A silencing
Source: Sci Rep. 2016 Aug 3;6:30792. doi: 10.1038/srep30792 (PMC4971516; doi:10.1038/srep30792)
Supplement: Supplementary Information [file srep30792-s1.pdf]

Supplementary material

Angelman syndrome-derived neurons display late onset of paternal *UBE3A* silencing

Jana Stanurova[1¶], Anika Neureiter[2¶], Michaela Hiber[1], Hannah de Oliveira Kessler[2], Kristin Stolp[2], Roman Goetzke[3], Diana Klein[4], Agnes Bankfalvi[5], Hannes Klump[2], Laura Steenpass[1,*]

[¶] Equal contribution

* Corresponding author

E-mail: laura.steenpass@uni-due.de

Phone: +49 201 723 4539

Fax: +49 201 723 5900

Extended and Supplementary Materials and Methods

Supplementary Figures and Tables

Extended and Supplementary Materials and Methods

Cell culture

All media, PBS and supplements were obtained from Life Technologies unless otherwise stated. Cells were split once a week in a ratio of 1:2 to 1:6 (feeder-dependent cells) or 1:6 to 1:12 (feeder-independent cells). For propagation, colonies were dissociated either using collagenase (feeder-dependent), or cell dissociation reagent (feeder-independent; STEMCELL Technologies), according to the manufacturer's recommendations. To yield single cell suspensions, cells were treated with Accutase for 10 minutes at 37°C and triturated. To support survival of single cells, 10 µM ROCK inhibitor (Y-27632, Calbiochem) was added to the medium until the first medium change.

Reprogramming of human dermal fibroblasts

Fibroblast cultures were established and reprogrammed using a lentiviral vector co-expressing the coding sequences of *POU5F1*, *KLF4*, *MYC* and *SOX2* together with the gene encoding tomato fluorescent protein (Fig. 5A). Vector particle production was performed as described. Briefly, expression plasmids encoding lentiviral gag-pol (pcDNA3.GP.CCC), HIV-rev (239_RSV_Rev) and the reprogramming vector backbone (pRRL.PPT.SF.OKSM.I.GFP.Pre) were transiently transfected into HEK293 cells together with pMDG for VSV-G pseudotyping. Culture supernatants were collected 24 and 48 hours post transfection. The vectors were concentrated by ultracentrifugation of the supernatants at 27,000 x g, 1.5 h, 4 °C and subsequently titrated on HT1080 cells. For reprogramming, primary fibroblasts cultures were kept at about 80 % confluency. Fibroblasts were detached by trypsinization, counted and transferred to a culture vessel at a density of 4000 cells / cm^2 in culture medium (DMEM high glucose, 10 % FCS, 50 U / ml Penicillin / Streptomycin, 1 % sodium pyruvate, 1 % glutamine, 1 % non-essential amino acids and 0.2 % β-mercaptoethanol). Transduction was performed by adding vector supernatant according to a multiplicity of infection of ten. Medium was exchanged 24 hours later. After flow cytometric cell sorting, cells were and plated at a density of 1,000 cells / cm^2 in a 6-well plate on

proliferation-arrested CF-1 feeder cells in KSR medium containing 10 µM ROCK inhibitor). Colonies were picked approximately four weeks post transduction and expanded on irradiated feeder cells in KSR medium.

Virus excision

Virus excision was mediated by direct protein transduction of Flp recombinase into iPSCs. For this purpose, vector containing supernatants were collected from HEK293 cells transfected with 5 µg of Flp_Gag_Pol plasmid (pcDNA3.MA.Prot.Flpo) mixed with 15 ug of wildtype Gag-Pol plasmid (MLV_SynGag) and 2 ug of pMDG-VSVG plasmid (pMDG) encoding the envelope protein. Vectors were concentrated by ultracentrifugation at 27,000 x g, 1.5 h, 4 °C. iPSCs were seeded as single cells at a density of $1x10^4$ cells / cm^2 onto Vitronectin-coated 6-well plates and cultured in mTesR1 containing 10 µM ROCK inhibitor. 50 µl of vector-containing supernatant was added with daily medium changes until colonies appeared (about 5 days). Single clones were isolated and expanded. Genomic DNA was isolated using the QIAamp DNA mini kit (Qiagen) and 100 ng were used as template for PCR amplification. PCR amplification was conducted as a three-primer PCR which amplified a product specific for the intact integrated virus genome (290 bp) and an internal control (170 bp). The internal control is a fragment of the ∆U3-region of the virus that is present before and after excision. Successful excision was confirmed by absence of the 290 bp amplification product.

Immortalization of fibroblasts

Primary fibroblasts of the patient’s skin biopsy showed insufficient proliferation and early onset of senescence. In order to maintain cells and prolong proliferation, fibroblasts were immortalized by lentiviral transduction of a SV40 large T / small T antigen fusion protein encoded by the plasmid pCL12SVT (a kind gift of Helmut Hanenberg, Indiana University, USA). Since selection of transduced cells is not possible with this construct, different volumes (2, 5, 10, 15, 20 and 40 µl) of virus supernatant were added to $2x10^5$ cells per well

in a 6-well plate. Transduced cells were selected by proliferation rate and survival. Immortalized patient fibroblasts were solely used for analysis of DNA methylation.

HLA typing

Isolated genomic DNA of iPSCs was used for high resolution HLA-A, -B and -C typing, using the Luminex Multiplex Technology together with commercially available sequence-specific oligonucleotides (High Definition LABType)[1]. Briefly, exons 2 and 3 of HLA class I alleles were amplified, biotinylated and subsequently hybridized to the sequence-specific, microbead-bound oligonucleotides. Bound oligonucleotides can be identified by the Luminex flow analyzer. HLA class I alleles with identical nucleotide sequences were grouped.

Alkaline phosphatase staining

iPSC colonies were stained for alkaline phosphatase activity using the Alkaline Phosphatase Detection Kit from Millipore as recommended by the manufacturer. The staining reaction was stopped after 15 minutes by washing with PBS and cell colonies were evaluated by phase contrast microscopy.

Immunofluorescence

For antibody stainings, cells were cultivated on feeder cells in standard 24-well cell culture dishes or in 24-well Lumox plates (Sarstedt). Cell colonies were fixed using 4 % paraformaldehyde for 15 min at room temperature. For staining of nuclear proteins, cells were permeabilized by incubation in 0.3 % (v / v) Triton X-100 for 5 min at room temperature. After washing and blocking in PBS with 3 % normal goat serum (serum of secondary antibody host species; Cell Signaling Technology), incubation with the primary antibody was conducted at 4 °C overnight. After washing with PBS, fluorescently labelled secondary antibody was applied for 2 hours at room temperature. Cells were counterstained with DAPI (200 µg / ml; diluted 1:1000 in PBS) and imaged on a Zeiss Axio Observer.D1 fluorescence

microscope using the Axiovision acquisition software from Zeiss. Antibodies and dilutions used are listed in Supplementary Table S7.

Flow cytometry

For FACS analysis of pluripotent cells cultured on feeder layer, feeder cell depletion was performed using Feeder Removal Microbeads (mouse) (Miltenyi Biotech) according to manufacturer's instructions. Briefly, cells were treated with Accutase (Life Technologies) to generate a single-cell suspension, which was passed through a 40 µm cell strainer and counted. After incubation with magnetic microbeads, the suspension was passed through an LS column (Miltenyi Biotech) and the flow-through (containing the iPSC cells) was concentrated by centrifugation and resuspended in FACS buffer (10 % fetal bovine serum in PBS). For each antibody staining, $1x10^5$ cells were used in a volume of 100-200 µl FACS buffer. Antibodies were used in a 1:100 dilution and incubated with the cell suspension for 20 min at 4 °C. After addition of 1 ml FACS buffer and centrifugation (700 x g, 5 min), cells were resuspended in 200 µl FACS buffer and analyzed on a FACS Aria (Becton Dickinson) using the program FACSDiva (Becton Dickinson). Isotype controls were applied for every antibody. Data analysis was done using Kaluza® software (Beckman Coulter). Antibodies are listed in Supplementary Table S7.

Karyotyping

Cells were cultured in 6-well plates. At 50 to 60 % confluency cells were treated with 0.1 µg / ml colcemid (Roche) for 3-5 hours in a humidified incubator at 37 °C. Cells were harvested using cell dissociation buffer and collected by centrifugation at 200 x g for 7 min. Hypotonic treatment in 0.4 % Hepes-KCl was performed for 20 min at 37 °C in a water bath. At the end of hypotonic treatment, 100 µl of freshly prepared fixative (3:1 methanol: acetic acid) were added and the suspension was mixed by inversion. Cells were collected by centrifugation at 200 x g for 7 min. 5 ml of fixative were added to the cell pellet and the suspension was incubated for 20 min at room temperature. After centrifugation, the pellet was resuspended in

500 µl fixative. Two drops were placed on a pre-chilled slide to test for chromosome quality using a microscope. For karyotype analysis, slides were baked overnight at 60 °C, digested with 0.25 % trypsin solution for a maximum of 5 min and stained with Giemsa (MerckMillipore) for 3.5 min. Chromosome analysis was done using a Zeiss Axioskop microscope and the Ikaros software (MetaSystems). Per sample eleven metaphases were counted and analyzed. To determine the origin of the marker chromosome in patient #H, XCyting chromosome paints (Metasystems) for chromosomes 12 and 17 were applied for fluorescence in situ hybridization according to manufacturer's instructions.

Neuronal differentiation

For positive selection of neural progenitor cells at day 13 magentic PSA-NCAM MicroBeads (Miltenyi Biotec) were used. In brief, a single cell suspension was passed through a 40 µm cell strainer (Biologix) and a maximum of $1x10^7$ cells was blocked in 60 µl blocking buffer (1% BSA in PBS) for 5 minutes at room temperature. Next, 20 µl of the magnetic beads were added and cells were incubated at 4 °C for 15 minutes. After addition of 2 ml blocking buffer and centrifugation, the pellet was resuspended in 500 µl induction medium and loaded onto an LS column placed into a magnetic separator (Miltenyi Biotec). The column was washed three times with expansion medium. After removal of the column from the magnetic separator, cells were resuspended in 5 ml expansion medium and seeded at a density of 4×10^5 / cm^2 on Matrigel-coated culture vessels. Culture was continued in expansion medium. Cells were passaged every three to four days as single-cell suspension and seeded at densities of 4×10^5 / cm^2 at the first four passages and afterwards at 1×10^5 / cm^2 on Matrigel-coated culture vessels. Terminal differentiation into neurons followed.

qRT-PCR and TaqMan human stem cell pluripotency arrays

RNA was prepared by cell lysis in Qiazol (Qiagen), chloroform extraction and subsequent processing of the aqeous phase through columns from the RNAeasy kit (Qiagen) according to the protocol. 500 ng DNaseI-treated RNA was reverse transcribed into cDNA using either

Quantitect Reverse Transcription Kit (Qiagen) or the GeneAmp RNA PCR Core Kit (Life Technologies). For qPCR, QuantiFastSYBR Green PCR Kit (Qiagen) was used with the amount equivalent to 10 ng of reverse transcribed RNA input and run on a CFX96 Real Time System C1000 thermal cycler (BioRad) with an annealing / extension temperature of 60 °C and 40 cycles. No-template and no-RT negative controls were included. Analysis was performed using the BioRad CFX manager 3.1 software. Plots were generated using the Origin Pro software (OriginLab). Expression of endogenous encoded genes was tested for using primers binding in the untranslated regions of the mRNA templates. The primer pair used for amplification of *SNHG14* was published by Runte et al, it locates about 65 kb downstream of *UBE3A* and spans an intron of about 1.7 kb[2]. This avoids amplification of fading *UBE3A* transcripts or of genomic DNA. The primers used for amplification of *UBE3A* have been published by Rougeulle et al, spanning exons 11 to 12. Expression was normalized to housekeeping genes *GAPDH* and *RPL13A* and then calibrated to the expression levels of hESCs H1, which were used as a standard. Primer sequences and product sizes are listed in Supplementary Table S8.

For TaqMan human stem cell pluripotency arrays (Life Technologies, catalog number 4385225), 500 ng DNaseI-treated RNA was reverse transcribed into cDNA and cDNA equivalent to 100 ng of input RNA was loaded into two reservoirs of the microfluidic cards by centrifugation. Arrays were run on an ABI PRISM 7900HT sequence detection system (Applied Biosystems, Life Technologies). Cycle threshold analysis was done using the ABI PRISM 7900HT sequence detection system software (SDS 2.1) of Applied Biosystems (Life Technologies). Heatmaps and cluster analysis of data was done using heatmap.2 in The R Project for Statistical Computing with default settings.

Deep bisulfite sequencing

Genomic DNA of cultured cells was prepared using standard alkaline lysis protocols. Bisulfite conversion of 500 ng of genomic DNA was performed using the Zymo EZ methylation Gold kit (Zymo) according to the manufacturer´s protocol. 1 µl of converted DNA was used in a

first round of PCR with Qiagen HotStart MasterMix and amplicon-specific primers, which carry a tag at their 5'-end. Using primers binding to this tag sequences and containing multiplex identifier (MID) barcodes and adapters for the 454 sequencing process were added to the amplicons in a subsequent PCR. Amplified DNA products were purified, enriched, pooled and processed for sequencing on the 454 GS Junior platform (Roche). Post-run data analysis included quality filtering using a special bisulfite filter and data processing using the Amplikyzer software. Overall percent methylation was calculated using the number of all methylated CpGs in all reads in relation to the total number of CpG called in all reads and is presented in Supplementary table S6.

Pyrosequencing and Epi-Pluri-Score analysis

1 µg of genomic DNA was bisulfite converted using the EZ DNA Methylation Kit (Zymo) and the three regions of interest (containing CpG sites cg 23737055 in *ANKRD46*, cg22247240 in *VRTN*, cg13083810 in *POU5F1*) were amplified by PCR. From this PCR product, a single-stranded DNA molecule was prepared using the PyroMark Q96 Vacuum Prep Workstation (Qiagen), which was then sequenced on the PyroMark Q96 ID System using a gene specific sequencing primer. Analysis was conducted with the PyroMark CpG SW1.0 software (Qiagen). As result of pyrosequencing, so-called β-values are obtained, ranging continuously from 0 (not methylated) to 1 (fully methylated). Values between 0 and 1 reflect the overall level of DNA methylation observed at one specific CpG site in a pool of cells. The Epi-Pluri-Score is calculated as the difference of β-value(*ANKRD46*) minus β-value(*VRTN*) and plotted against the β-value measured for *POU5F1*.

Southern blot analysis

Genomic DNA was prepared by alkaline lysis and 20 µg of genomic DNA were digested either with BamHI or EcoRV overnight and run on 0.8 % (w / v) agarose gels at 60 V for 5 to 6 h. Gels were denatured in 0.5 M NaOH / 1.5 M NaCl solution, twice for 30 min. Wet blotting to positive-charged nylon-membranes (Amersham Hybond-XL, GE Healthcare) was

performed for at least 16 h in denaturing solution. Blots were hybridized with a wPRE fragment isolated from the reprogramming vector by EcoRV digestion. The probe was labeled with α-^{32}P-dCTP using the Megaprime kit (GE Healthcare) and purified using the QIAquick Nucleotide Removal Kit (Qiagen). Hybridization was performed at 65 °C overnight in Church buffer (7 % SDS, 1 mM EDTA, 0.5 M Na_2HPO_4 / NaH_2PO_4, pH 7.2). After stringent washing in Church buffer at 65 °C for at least 30 min twice, blots were exposed to film at -80 °C and developed after one to ten days.

Western blot analysis

Protein lysates of 1 x 10^6 were prepared in 100 µl NP-40-lysis buffer (500 mM NaCl, 1 mM EDTA, 20 mM Tris, 0.5% (v/v) NP-40, protease inhibitor cocktail, pH 8), incubated on ice for 30 min with vortexing every 10 min. After centrifugation for 20 min at 4 °C at 14.000 rpm, the supernatant was transferred into a new tube and protein concentration was determined using the BCA Protein Assay Kit from Thermo Fisher. 10 µg protein extract was resolved on a standard denaturing 10 % SDS gel at 45 mA for 100 min. Proteins were transferred to PVDF-membrane by wet blotting in transfer buffer (25mM Tris, 192 mM glycine, 20% (v/v) methanol) at 75 V for 100 min. After membrane blocking in 5% milk powder for 1h, incubation of the first antibody was performed in 5% milkpoweder in TBST (20mM Tris, 150 mM NaCl, 0.1 % Tween 20, pH 7.5) at 4 °C over night with agitation. After membrane washing for 3 times in TBST, incubation with the secondary antibody was performed in TBST for 1h at room temperature with agitation. Detection was performed using the Super Signal West Femto Maximum Sensitivity Substrate from Thermo and signals were recorded with a Fusion FX7 device. Incubation with the UBE3A-antibody was performed first and followed by incubation with the α-tubulin antibody after stripping of the membrane. Stripping was performed in Restore Western Blot solution from Thermo.

Supplementary references

1 Heinemann, F. M. HLA Genotyping and Antibody Characterization Using the Luminex Multiplex Technology. *Transfus Med Hemother* **36**, 273-278, doi:10.1159/000228834 (2009).

2 Runte, M. *et al.* The IC-SNURF-SNRPN transcript serves as a host for multiple small nucleolar RNA species and as an antisense RNA for UBE3A. *Hum Mol Genet* **10**, 2687-2700 (2001).

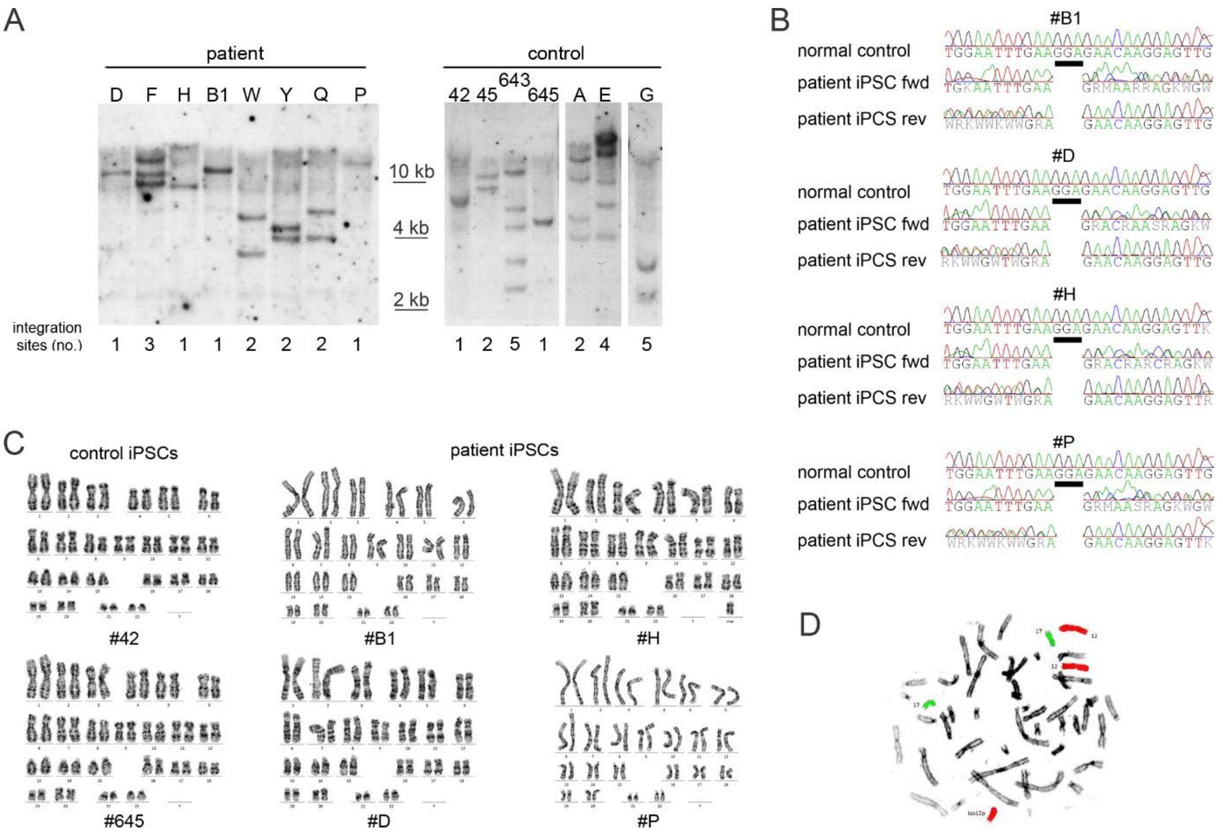

Fig. S1: Characterization of iPSCs. A) Southern blot of patient and control iPSC clones. DNA was digested with BamHI (left) or EcoRV (right); the probe used for hybridization binds to the integrated lentiviral vector used for reprogramming. The number of vector integration sites is indicated below the blots. **B)** Sanger sequencing of three patient iPSC clones. Forward and reverse sequencing of *UBE3A* exon 4 (accession NM_130838) is shown for patient clones #B1, #D, #H and #P. Top: sequence of a normal control person. The deleted triplet, encoding amino acid glycine at positon 538 is indicated by the black bar. The sequence becomes ambiguous at the deletion. **C)** Normal female karyotypes of control #42 and #645 (left), and patient #B1, #D and #P (right). Patient #H has an aberrant karyotype showing one additional marker chromosome (lower right). **D)** Whole chromosome painting of a metaphase spread of patient #H using probes for chromosomes 12 (red) and 17 (green) identifies the marker chromosome as an isochromosome 12p.

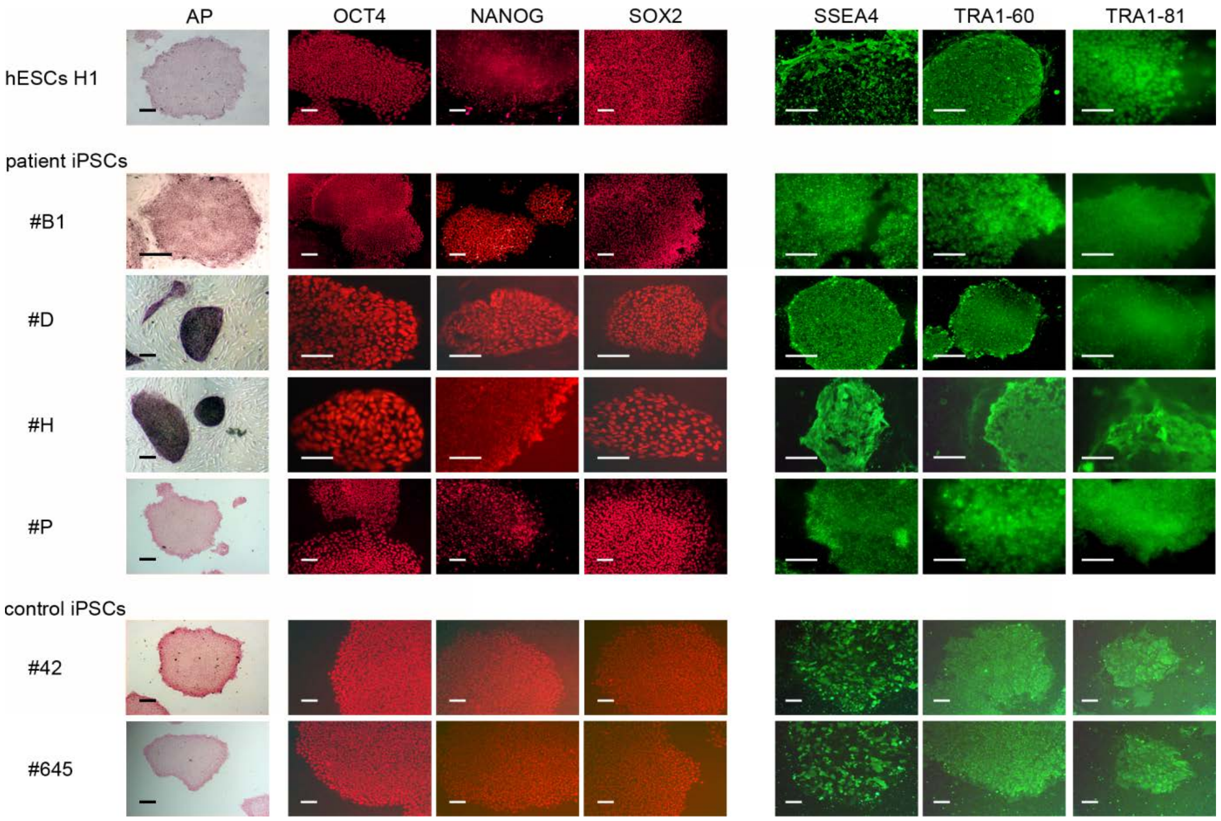

Fig. S2: Alkaline Phosphatase and immunofluorescent staining of iPSCs. Expression of surface alkaline phosphatase (AP) is indicative for pluripotent cells. Staining of four patient iPSC clones and two control iPSC clones is shown. Immunofluorescence of nuclear (OCT4, NANOG, SOX2; red) and surface (SSEA4, TRA1-60, TRA1-81; green) antigens is shown for four patient iPSC clones (#B1, #D, #H, #P) and two control iPSC clones (#42 and #645). Note that images of #D are also shown in Fig. 2 of the main paper. Positive staining indicates pluripotency. The hESC H1 line was used for reference. The scale bar represents 100 µm.

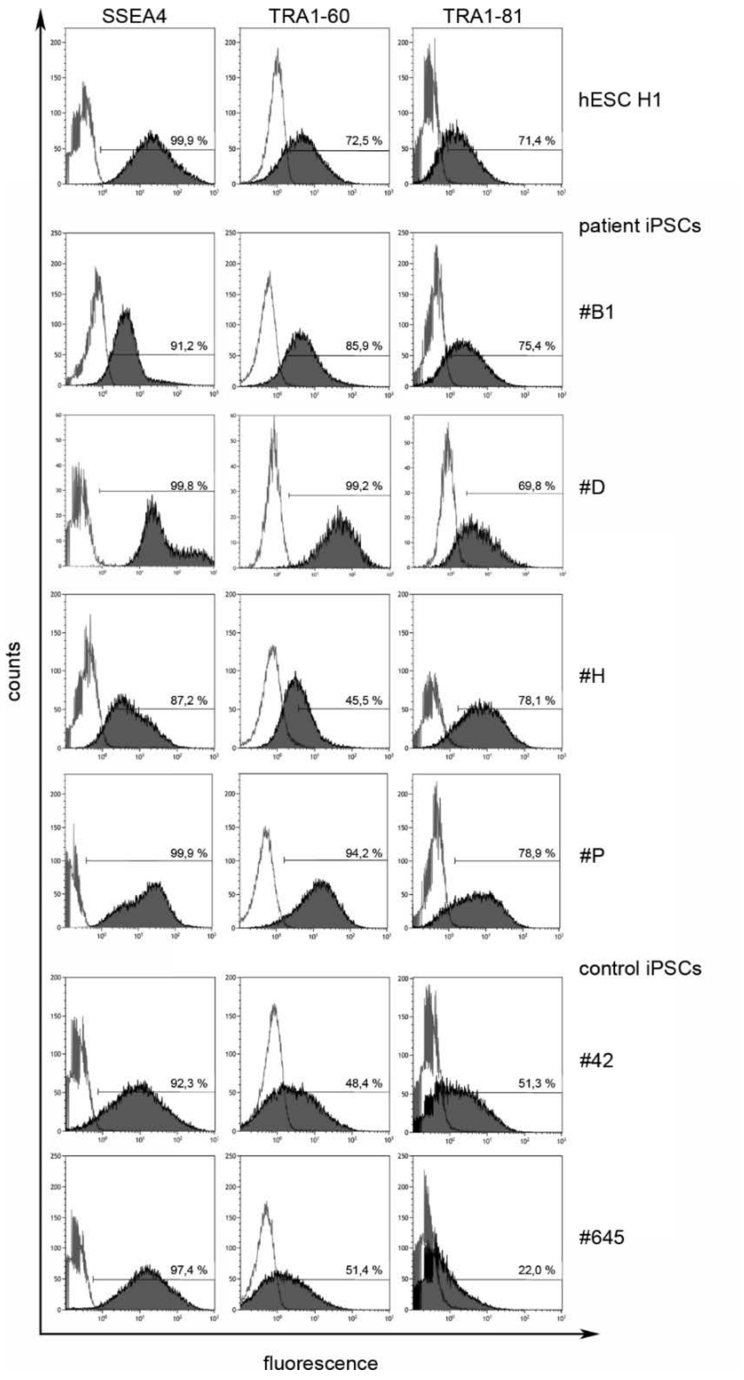

Fig. S3: Flow cytometry of iPSCs. Expression of pluripotency-associated surface antigens SSEA4, TRA1-60 and TRA1-81 was analyzed by flow cytometry. Isotype controls with an unrelated antibody are shown in white, staining with specific antibodies in grey. Positive staining results in a shift of the cell population to the right. Quantification is given as percentage of cells in the designated region. hESC H1 was included for comparison.

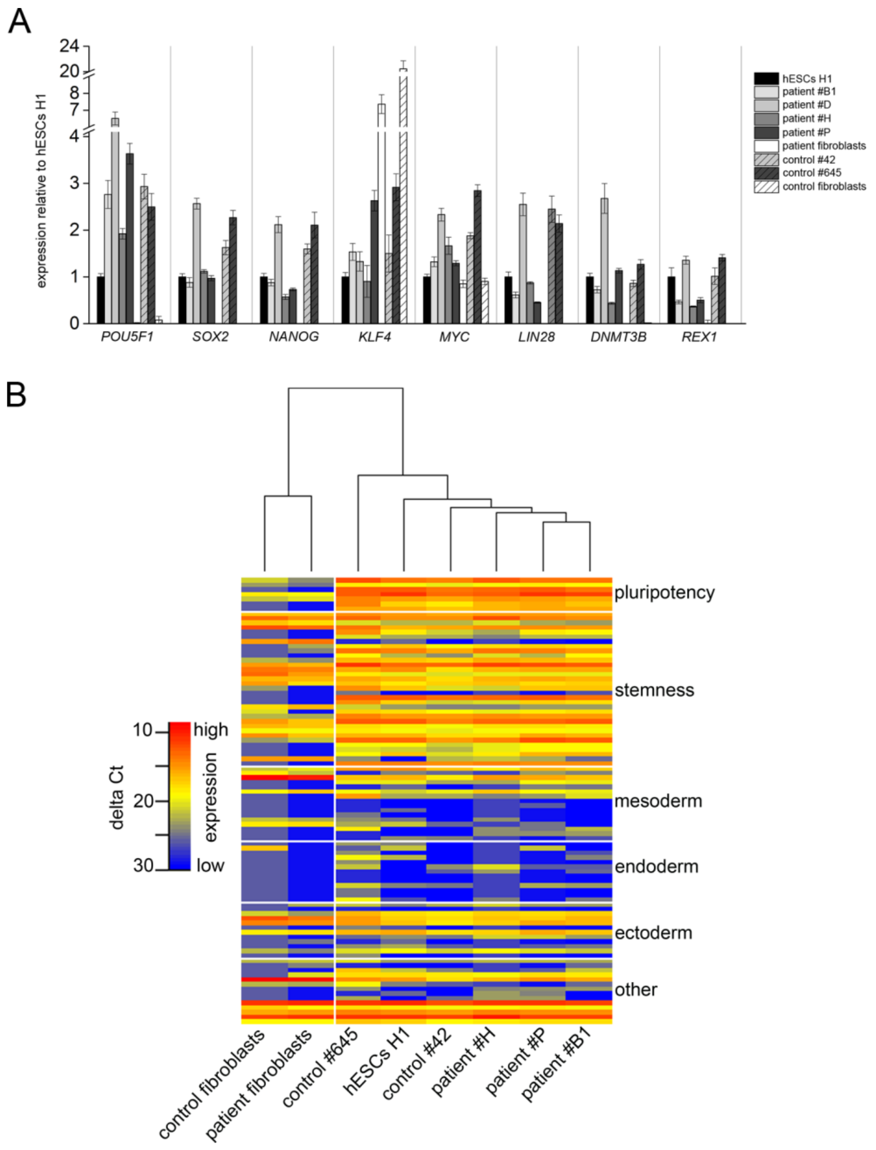

Fig. S4: Expression profiling of pluripotency-associated marker genes. A) Expression of eight pluripotency-associated marker genes was analyzed by quantitative qPCR in iPSCs and parental fibroblasts. Expression is displayed relative to gene expression in hESC H1, indicating comparable levels. Standard deviation of three technical replicates is given. No-template and no-RT controls were performed and gave no result. **B)** Gene expression measured by TaqMan human srem cell pluripotency arrays was calculated relative to 18S rRNA (ΔCt) showing low expression in blue and high expression in red. Results are presented as heatmap of a cluster analysis, which was calculated using heatmap.2 in The R Project for Statistical Computing with default settings. Genes are ordered into groups as indicated on the right. Parental fibroblasts used for reprogramming cluster separately from pluripotent hESCs H1 and generated AS_Δ3 iPSCs.

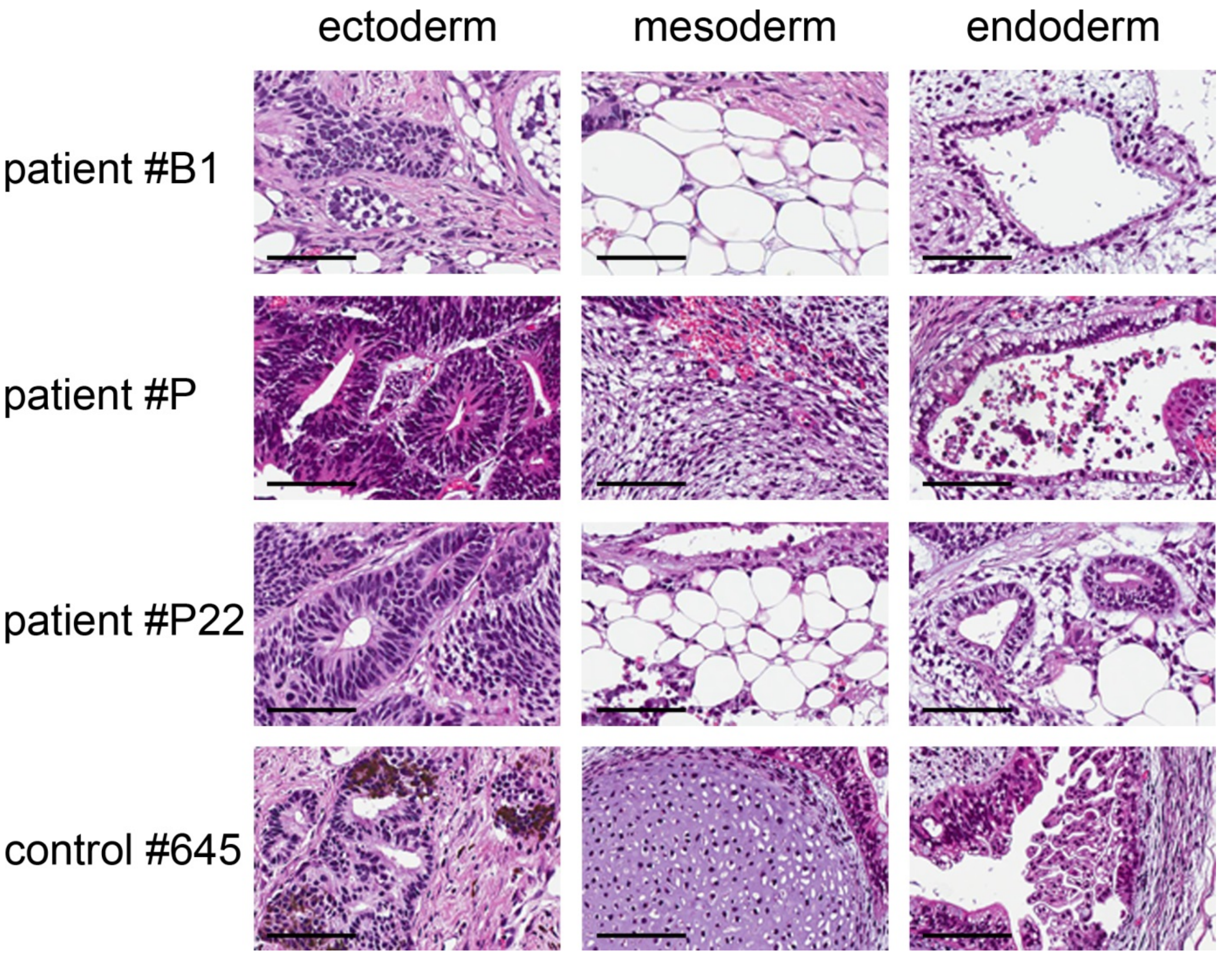

Fig. S5: Teratoma formation in patient and control iPSCs H&E staining of immature teratomas derived from iPSCs of patient #B1, #P and #P22 (virus excised) and control #645 shows presence of derivatives of all three germ layers. Ectoderm: neural rosettes; in control #645 with neuromelanin pigmentation. Mesoderm: fat cells (#B1; #P22), blood vessels and loose immature mesenchyme (#P) and immature cartilage (#645). Endoderm: glandular structures with columnar cells showing subnuclear vacuolization typical for clear cells. The scale bar represents 100 µm.

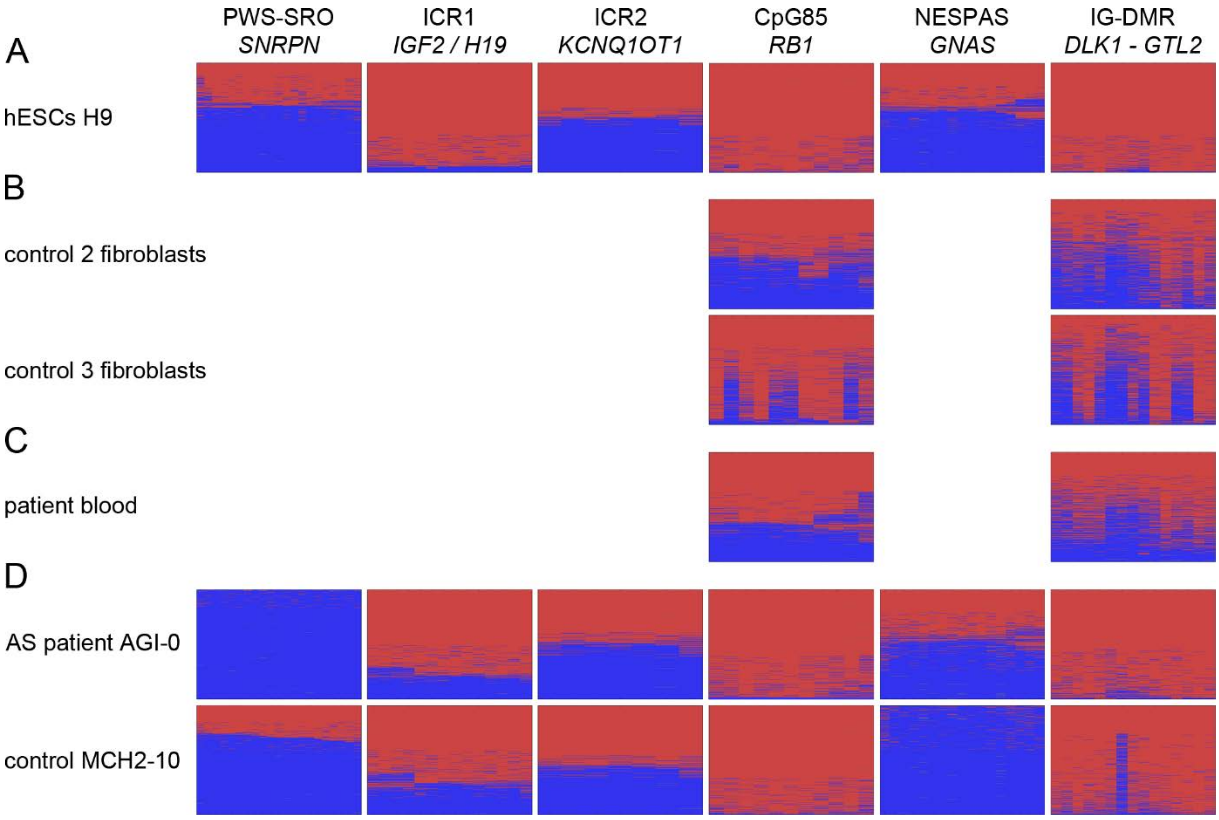

Fig. S6: DNA methylation at DMRs. DNA methylation at six gametic imprinted DMRs was analyzed by deep bisulfite amplicon sequencing. Results are displayed as methylation heatmaps with individual CpG sites in columns and reads in rows. Red: methylated, blue: non-methylated. **A)** hESC H9 at later passage shows gain of methylation at ICR1, CpG85 and the IG-DMR. **B)** Methylation of CpG85 and the IG-DMR was analyzed in fibroblast samples of two additional healthy control persons. Control 2 shows normal 50 % methylation at CpG85, but an increase of methylation at the IG-DMR. Control 3 has increased levels of methylation at both DMRs. **C)** Analysis of CpG85 and the IG-DMR in blood of the patient showed differential methylation at CpG85, but an increase at the IG-DMR. **D)** Analysis of methylation in iPSC lines of a patient with AS (AGI-0) and a normal control person (MCH2-10) obtained from S. Chamberlain (University of Conneticut Health Center, USA). Line AGI-0 was derived from an AS patient with a large deletion including the PWS-SRO on the maternal chromosome. As expected, from the remaining paternal allele only unmethylated reads were obtained. Both lines show a gain of methylation at ICR1. Both lines also exhibit a gain of methylation at CpG85 and the IG-DMR. In addition, MCH2-10 shows a loss of methylation at NESPAS.

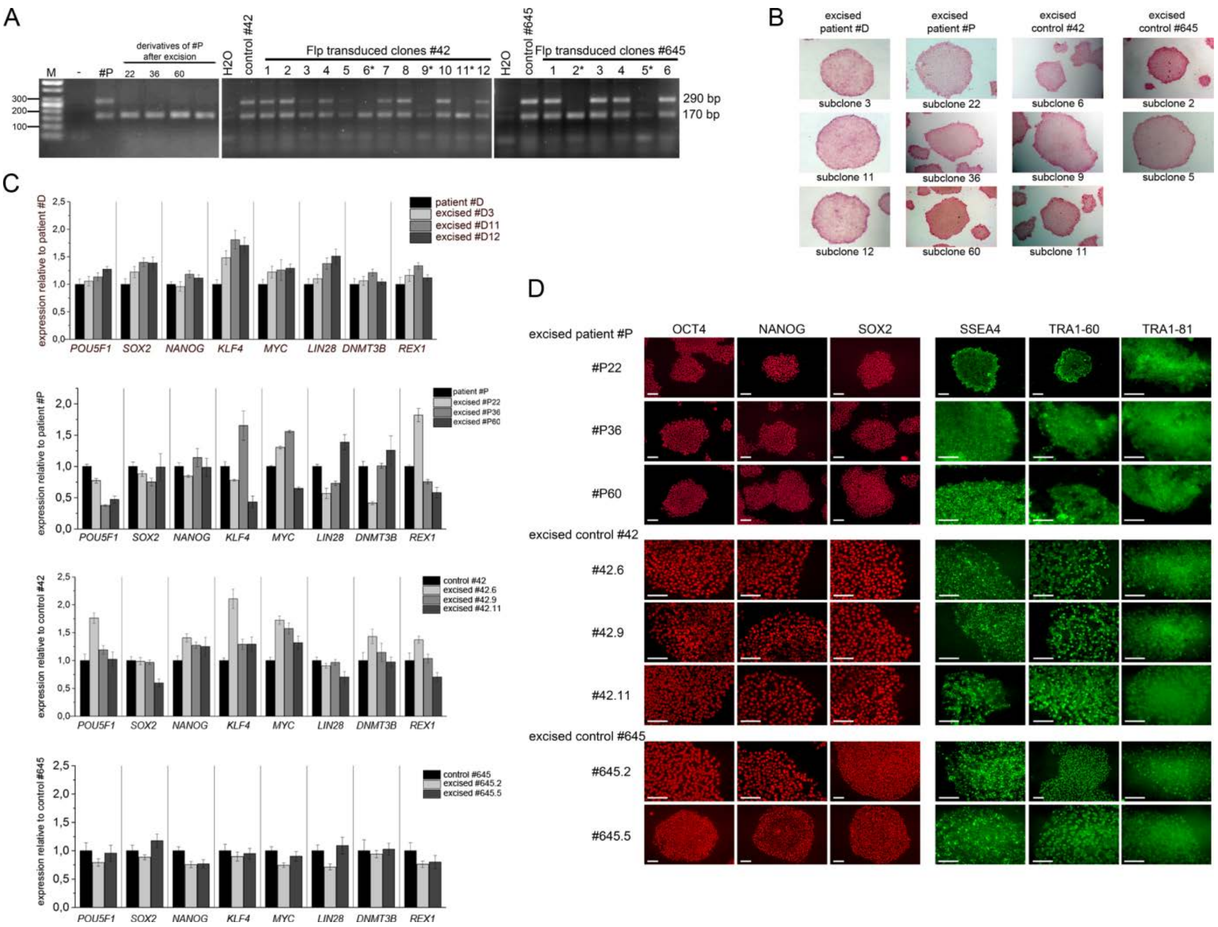

Fig. S7: Virus excision by Flp recombination. Virus excision was performed in patient #D and #P and control #42 and #645. **A)** Determination of successful excision by PCR. The larger product of 290 bp corresponds to the integrated virus, its absence indicates virus excision. The smaller product of 170 bp serves as internal control for successful PCR. **B)** Positive staining for alkaline phosphatase expression in all clones. Images were taken at 100x magnification. **C)** Expression analysis of eight pluripotency-associated marker genes in excised subclones as analyzed by qPCR. Expression is shown relative to levels obtained for parental iPSCs. Assays confirm the maintenance of pluripotency in iPSCs after excision of the integrated viral vector by Flp recombination. No-template and no-RT controls were performed and gave no result. **D)** Positive immunofluorescence for endogenous nuclear antigen expression (OCT4, NANOG, SOX2) and surface antigens (SSEA4, TRA1-60, TRA1-81) after virus excision. The scale bar represents 100 µm.

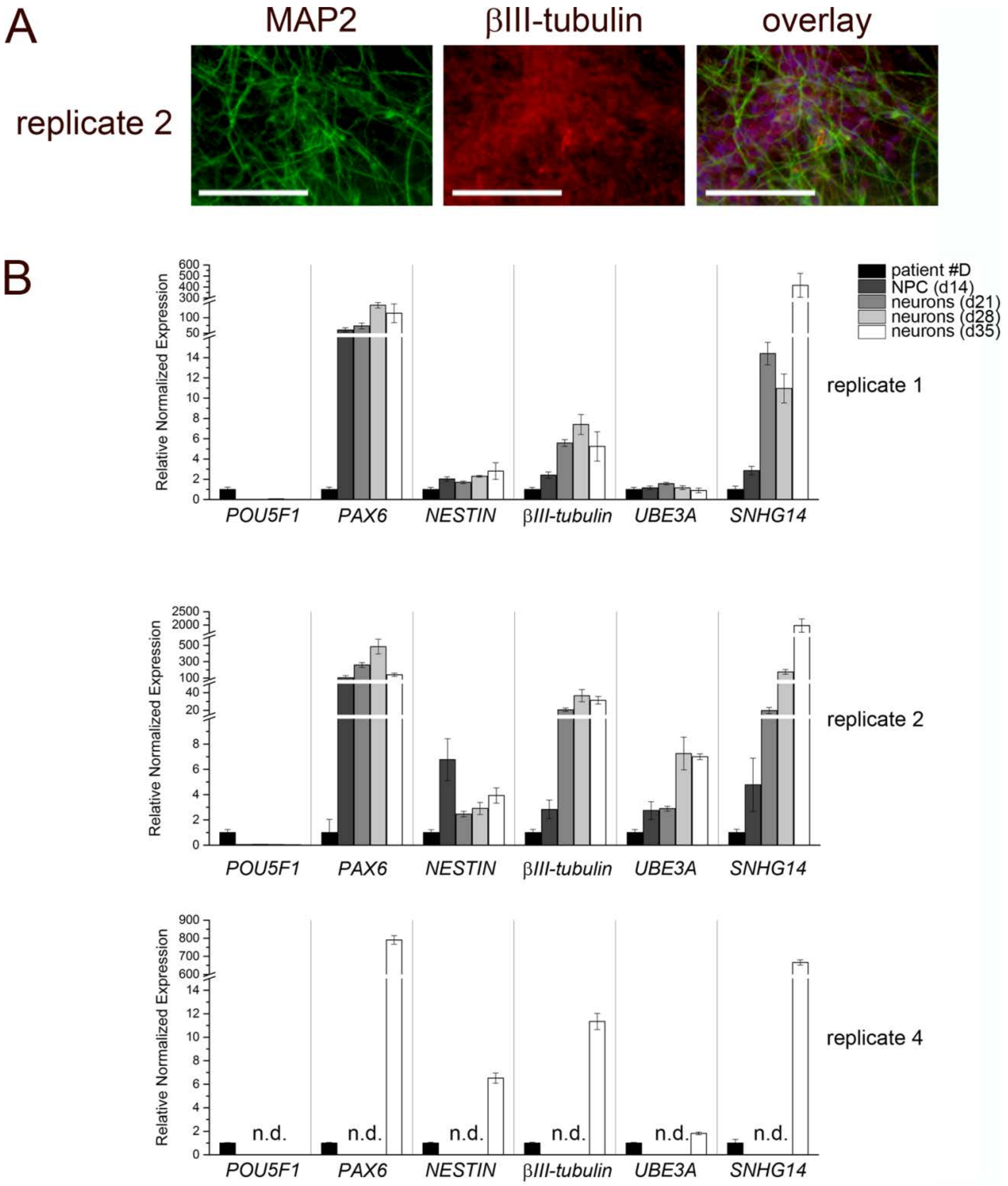

Fig. S8: Differentiation of patient #D iPSCs into neurons. Results of additional biological replicates are shown. **A)** Immunofluorescent staining for neuronal markers MAP2 and βIII-TUBULIN indicate differentiation of patient #D iPSCs into mature neurons. The scale bar indicates 100 µm. **B)** Analysis of marker gene expression showed upregulation of neuronal markers *PAX6*, *NESTIN* and *βIII-TUBULIN*. Upregulation of *UBE3A* and *SNHG14* was reproducibly observed. No-template and no-RT controls were performed and gave no result. n.d.: not determined.

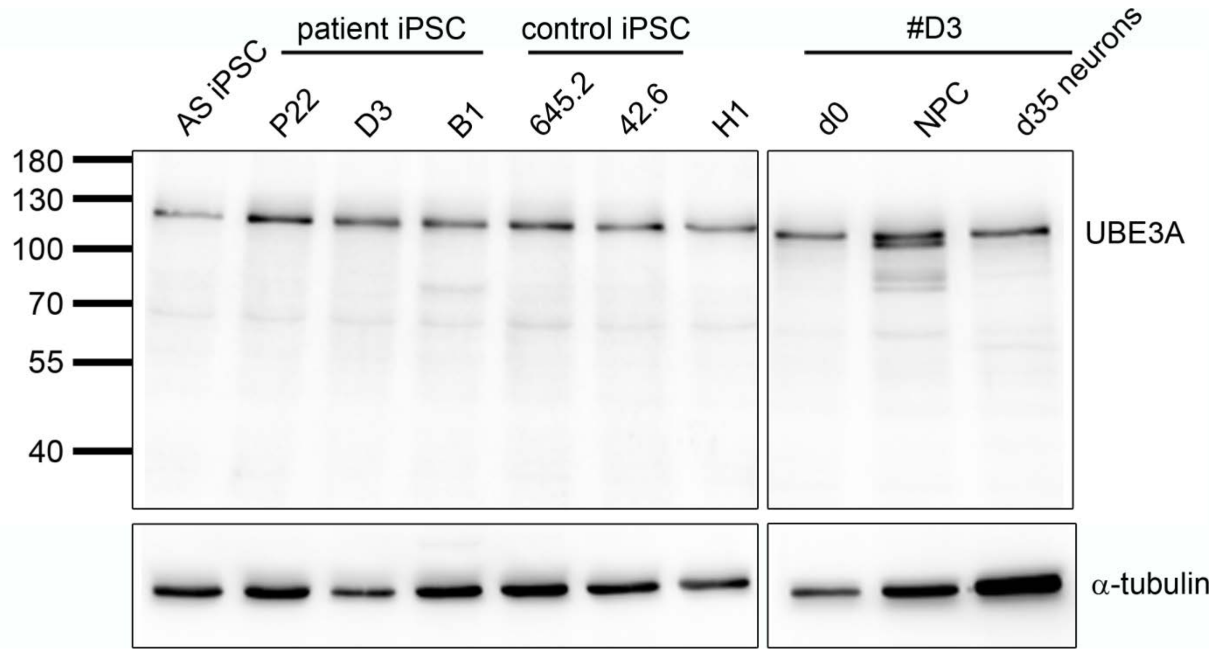

Fig. S9: Stable expression of UBE3A protein in iPSCs and neurons. Steady state amounts of UBE3A protein were observed during neuronal differentiation of patient iPSCs (#D3) by immunoblotting of total cell lysates (right panel). Undifferentiated AS iPSCs with a large deletion on the maternal allele (Chamberlain et al, 2010), three patient and two control iPSC clones and the reference hESC H1 were used for comparison (left panel). AS iPSCs express *UBE3A* only from the paternal allele, resulting in decreased amounts of UBE3A protein. α-tubulin was used as loading control. We have not yet identified the nature of the additional band visible only at the NPC stage of neuronal differentiation.

Table S1: Reprogramming efficiencies

Fibroblasts	Seeded	Clones isolated	Efficiency	Efficiency in %
**Patient**	15,000	8	8 in 15,000	0.05
**Patient**	555,000	30	30 in 555,000	0.005
**Healthy control**	345,000	28	28 in 345,000	0.008
**Healthy control**	968,000	113	113 in 968,000	0.01

Table S4: Teratoma formation

Cells of each iPSC clone indicated were injected into both flanks (left and right) of immunodeficient NMRI nu/nu mice. Two animals per cell line were used. Teratoma formation occurred with an efficiency of 100 % for all iPSC clones used but the growth rate differed. Weight (W) of tumors is given in grams (g). Tumors were explanted at critical size, when skin lesions appeared or after 67 days.

cells injected	side	W (g)	days to explantation
**hESC H1**	right	0.628	30
	left	0.020	30
**hESC H1**	right	0.244	37
	left	0.267	37
**patient #B1**	right	0.404	30
	left	0.015	30
**patient #B1**	right	0.295	30
	left	0.462	30
**patient #D**	right	0.098	67
	left	0.113	67
**patient #D**	right	0.1	67
	left	0.203	67
**patient #P**	right	0.241	32
	left	0.148	32
**patient #P**	right	0.385	47
	left	0.530	47
**patient #P22**	right	0.119	24
	left	0.629	24
**patient #P22**	right	0.083	24
	left	0.584	24
**control #645**	right	0.152	60
	left	0.090	60
**control #645**	right	0.389	60
	left	0.125	60

Table S5: Chromosomal regions analyzed for DNA methylation and the Epi-Pluri-Score

Chromosomal regions are listed according to the UCSC browser, genome build hg19

DMR / CGI	locus / gene	no. of CpG sites	chromosomal region
ICR1	*IGF2 / H19*	14	chr11:2021080-2021248
ICR2	Beckwith-Wiedemann locus	7	chr11: 2721560- 2721680
CpG85	*RB1*	11	chr13:48,893,505-48,893,690
IG-DMR	*DLK1 - MEG3*	15	chr14: 101277219-101277485
SNRPN	Prader-Willi / Angelman locus	21	chr15:25200011 -25200250
NESPAS	*GNAS*	17	chr20:57429210- 57429446
POU5F1	*POU5F1*	10	chr6:31,138,276-31,138,507
NANOG	*NANOG*	6	chr12:7,941,603-7,941,810
cg23737055	*ANKRD46*	1	chr8:100,559,768-100,559,941
cg22247240	*VRTN*	1	chr14:74,348,428-74,348,610
cg13083810	*POU5F1*	1	chr6:31,171,307-31,171,723

Table S7: Antibodies

Antibody	Supplier	Cat#	dilution
**Flow cytometry**			
PE anti-human TRA1-60-R	Bio Legend	330610	1:100
PE mouse IgM, isotype control	Bio Legend	401611	1:100
Alexa Fluor 488 anti-human TRA1-81	Bio Legend	330710	1:100
Alexa Fluor 488 mouse IgM, isotype control	Bio Legend	401617	1:100
Alexa Fluro 647 anti-human SSEA4	Bio Legend	330408	1:100
Alexa Fluor 647 mouse IgG3, isotype control	Bio Legend	401321	1:100
**Immunofluorescence**			
Stem Light Pluripotency kit	Cell Signaling Technology	9656S	1:200
OCT4 anti-human	STEMCELL Technologies	60093	1:1000
PAX6 anti-human	STEMCELL Technologies	60094	1:500
MAP2	Sigma Aldrich	M9942	1:300
βIII-TUBULIN	Life Technologies	PA-46430	1:300
**Secondary antibodies**			
Alexa Fluor 488 anti-mouse IgG	Life Technologies	A11001	1:800
Alexa Fluor 555 anti-rabbit IgG	Cell Signaling Technology	4413S	1:1000
Alexa Fluor 488 anti-rabbit IgG	Life Technologies	A11008	1:800
Alexa Fluor 488 anti-mouse IgG	Life Technologies	A11001	1:800
Alexa Fluor 488 anti-mouse IgG	Cell Signaling Technology	4408S	1:1000
**Western blot**			
anti-UBE3A	Becton Dickinson	611416	1:500
anti-α-tubulin	Sigma Aldrich	T5168	1:2000
donkey anti-mouse HRP	Dianova	715-036-150	1:10.000

Table S8: Primer sequences

Underlined sequences are tags for amplification with MID primers. The multiplex identifier (MID) sequences for barcoding of amplicons are in red, the key sequences for quality control are in blue.

Name	Sequence 5'->3'	Product size
**qRT-PCR pluripotency**		
OCT4_F	gaaggtgaagttcaatgatgctg	
OCT4_R	attcccatccctacctcagtaac	139
SOX2_F	agtatcaggagttgtcaaggcag	
SOX2_R	tcctagtcttaaagaggcagcaa	79
KLF4_F	ctaaatgatggtgcttggtgagt	
KLF4_R	ggtcataaatgttgatcggaagac	124
MYC_F	tcctgagcaatcacctatgaact	
MYC_R	ttgaggcagtttacattatggc	110
LIN28A_F	ttgaggagcaggcagagtgg	
LIN28A_R	tgcatttggacagagcatgg	162
NANOG_F	acctcagctacaaacaggtgaag	
NANOG_R	atccctggtggtaggaagagtaa	156
DNMT3B_F	ggatgtttgagaatgttgtagcc	
DNMT3B_R	gattacactccaggaaccgtga	72
REX1_F	agctgaaacaaatgtactgaggct	
REX1_R	ctccaggcagtagtgatctgagta	127
GAPDH_F	tgcaccaccaactgcttagc	
GAPDH_R	ggcatggagtgtggtcatgag	87
RPL13A_F	ccatcgtggctaaacaggagt	
RPL13A_R	aggaaagccaggtacttcaactt	112
**qRT-PCR neuronal differentiation**		
UBE3A_F	ctcttcttgcagtttacaacgg	
UBE3A_R	cttgagtattccggaagtaaaagc	152
SNHG14_RT17_F	cttgagtattccggaagtaaaagc	
SNHG14_RT17_R	cttgagtattccggaagtaaaagc	120
PAX6_F	aacagacacagccctcacaaaca	
PAX6_R	cgggaacttgaactggaactgac	275
NESTIN_F	ggaagagaacctgggaaagg	
NESTIN_R	cttggtccttctccaccgta	122
βIII-TUBULIN_F	ctcaggggcctttggacatc	
βIII-TUBULIN_R	caggcagtcgcagttttcac	160
GAPDH_F	tgcaccaccaactgcttagc	
GAPDH_R	ggcatggactgtggtcatgag	87
**Sequencing of UBE3A deletion in exon 4**		
UBE3A_100bp_fw	cctgcagacttgaagaagca	
UBE3A_100bp_r	cctccacaaccagctgaaa	100
**Detection of reprogramming vector excision**		
Flpo_1_F	cgagtcggatctccctttgggc	290
Flpo_2_F	tggaagggctacgtagctagc	170

Flpo_3_R	ggttccctagttagccagagagc	
**Bisulfite sequencing**		
H19-in-Ftag	cttgcttcctggcacgagggtayggaattggttgtagttgtgg	
CTCF-RM13_R_CTCF6	caggaaacagctatgacatatcctattcccaaataacccc	204
LIT1-Not1-F-tag	cttgcttcctggcacgagtttataggtttttatat**y**gagggtttatagtag	
LIT1-Not1-R-M13	caggaaacagctatgacaaataaac**yr**aaaacac**r**aaccaattctctac	156
IG-DMR-Ftag	cttgcttcctggcacgaggtttattgggttgggttttgttag	
IG-DMR-RM13	caggaaacagctatgacaccaattacaataccacaaaattac	302
NESPAS_F_neu_Bis	cttgcttcctggcacgaggtagtagtttttggatggagatttt	
NESPAS_R_neu_Bis	caggaaacagctatgacaaaaaaatacttttcctccctcc	272
SNRPN_Ftag	cttgcttcctggcacgagggagggagttgggatttttgtattg	
SNRPN_RM13	caggaaacagctatgacccccaaactatctcttaaaaaaaaccacc	275
RB1_CpG85_Ftag_1	cttgcttcctggcacgagtttggatggtttttttag	
RB1_CpG85_RM13_1	caggaaacagctatgacaacaaaaaaacaaaaacaacc	221
Bis_hOCT4_R2a_Ftag	cttgcttcctggcacgaggggggttagaggttaaggttagtg	
Bis_hOCT4_R2a_RM13	caggaaacagctatgacattcctaaccctccaaaaaaac	267
NANOG_R1_F_Stricker	cttgcttcctggcacgagatgttggttaggttggttttaaatt	
NANOG_R1_R_Stricker	caggaaacagctatgaccccaacaacaaatacttctaaattcaccac	242
AutMID1 (example)	cgtatcgcctccctcgcgccatcagacgagtgcgtcttgcttcctggcacgag	
ButMID1 (example)	ctatgcgccttgccagcccgctcagacgagtgcgtcaggaaacagctatgac	amplicon size
**Epi-Pluri-Score analysis / pyrosequencing**		
ANKRD46_for	biotin-aggggaggggttagataggggtag	
ANKRD46_rev	ccacaattttaatactttccctaattcaaaac	173
ANRD46_seq	cacaattttaatactttccc	
C14ORF115_for	biotin-gattttttgggggagtggtttaagt	
C14ORF115_rev	ccaccttccaacctaaaacatttaaatcacc	182
C14ORF115_seq	ccaaaaccaatttcatatc	
POU5F1_for	gatttttggtattgtgtttttaggggttagtta	
POU5F1_rev	biotin-taaaacccaatcaatccaaaatctaatccc	416
POU5F1_seq	tagtttttaaatttattgaatg	
